# LIFE AS A JOURNEY: A VIEW OF LIFE AS A JOURNEY MODERATES THE RELATIONSHIP BETWEEN SUBJECTIVE AGE AND SUBJECTIVE HEALTH

**DOI:** 10.1093/geroni/igz038.1702

**Published:** 2019-11-08

**Authors:** Ga-Eun (Grace) Oh

**Affiliations:** Open University of Hong Kong, Kowloon, Hong Kong

## Abstract

Previous research has shown the relationship of subjective age and health status: feeling younger than one’s age is correlated with better health outcomes including both subjective and objective measures. This research investigates how the view of life as a journey might moderate the relationship between subjective age and subjective health. A view to look at life as a journey is a common metaphor to view life as an ongoing process. Prior work has suggested that people who went through difficult situations successfully tend to construe their life experience as a journey. This suggests that thinking of life as a journey might help people cope better with their negative experiences in general such as feeling older. Thus, we investigate to see if believing ‘life as a journey’ can buffer against the negative effect of feeling older on subjective health perception. To test this, we collected the data from American participants (N = 724) of various ages. The results showed that more life was viewed as a journey, smaller the detrimental effect of subjective age on subjective health. Although feeling older generally reduced subjective health, this negative effect of feeling older was smaller among those who thought life as a journey. This research suggests that thinking life as a journey might be used to reduce the negative impact of older subjective age on health perceptions.

